# Which incision is better for Lewis to Brown Norway rat liver transplantation, transverse or midline?

**DOI:** 10.1016/j.heliyon.2023.e18213

**Published:** 2023-07-14

**Authors:** Gaofeng Tang, Huibo Zhao, Guoyong Chen, Shaotang Zhou

**Affiliations:** 6th Hepatopancreaticobiliary Surgery of Henan Provincial People's Hospital, People's Hospital of Zhengzhou University, 7 weiwu Road, Jinshui district, Zhengzhou, Henan, 450003, China

**Keywords:** Orthotopic liver transplantation, Rat, Self-biting

## Abstract

Orthotopic rat liver transplantation (OLT) is a complex microsurgical procedure extensively applied to basic science, myriad complications can occur, but incision-related self-biting has not been reported after OLT. For the project of tolerance induction through stem cells, we performed OLT from Lewis to Brown Norway (BN) rats as an acute rejection model and divided the study was into the transverse incision group (n = 15) and midline incision group (n = 22), while cyclosporine A was subcutaneously injected for 10-day immunosuppression use, lidocaine cream was used for pain-relieving. The recipient survival and wound status were the primary endpoint of this study. For the transverse incision group, 30-day survival rate was 40% (6/15), self-biting occurred in 13 cases in 7–39 days, the degree 1 of biting occurred in 1 cases, the degree 2 in 2 cases. The degree 3 in 10 cases, which caused death or euthanasia, the self-biting rate was 86.7% (13/15), For the midline incision group, 30-day survival rate was 100% (22/22), the degree 1 of self-biting occurred in 3 cases, no severe self-biting occurred. There were significant differences for survival (*p* = 0.0003) and for self-biting rate (*p* < 0.01) between two groups. In conclusion, incision-related self-biting behavior occurs due to incisional injury, the transverse incision is severely pain-causing; the midline one is effective to avert occurrences.

## 1 Introduction

Rat OLT is well accepted as the small animal model in studying ischemia-reperfusion injury, immunology and liver regeneration after its development by Lee and his colleagues [[1], [2], [3], [4]], this procedure is still challenging and complications occur intra- and post-operation like blood loss, thrombosis and graft dysfunction etc [[5], [6], [7], [8]]. As basic sciences progress, long-term survival for recipient rats is required in chronic rejection and tolerance induction for immunology and transplant medicine, surgical performances are significant for this model. For incision selection, many novices first prefer to transverse incision, which facilitates rapid surgical success but does greater damage to recipients, and incision-related events will occur. The incision-related outcomes after rat liver transplantation are not reported in the literature. Here we first presented these results after liver transplantation with different incisional selections.

### 1.1 Methods and materials

Male Lewis and BN rats (weighing 180–380 g) respectively served as donors and recipients purchased from Beijing Vital River Laboratory Animal Technology Corporation. We performed rat OLT from Lewis to BN for tolerance induction. In the beginning, microsurgeon preferred to the transverse incision for rapid success of OLT and encountered incisional complications, the midline incision was selected later, so the transverse incision group (n = 15) and midline group (n = 22) were used for our study. The rats were cared in a temperature and light-controlled house, the standard food and bottle water was freely accessible; they were fasted 12 h before the operation. All experimental protocols were approved by Committee of Henan Provincial People's Hospital and all experiments were carried out in compliance with the ARRIVE guideline and the standards for animal use and care set by the Institutional Animal Care Committee of People's Hospital.

### 1.2 Surgical procedure

**Fig. 1:**
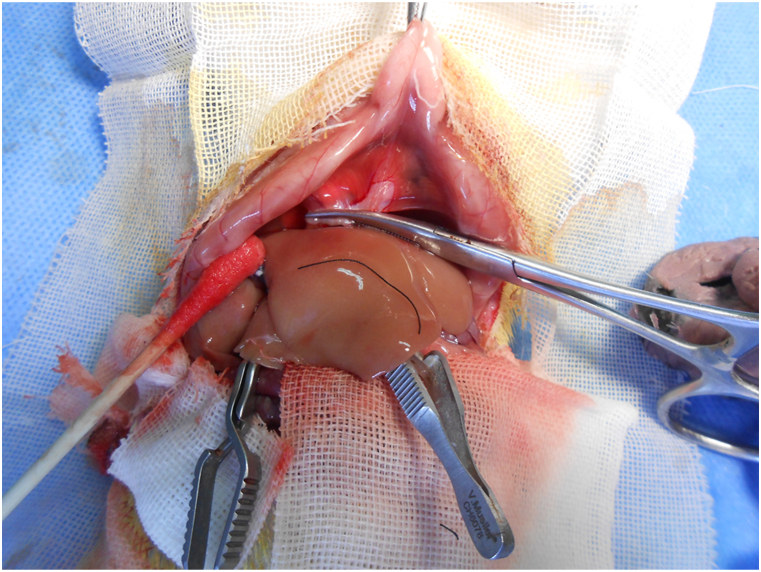
Transverse incision for OLT. It was good for exposure and performance.

**Fig. 2:**
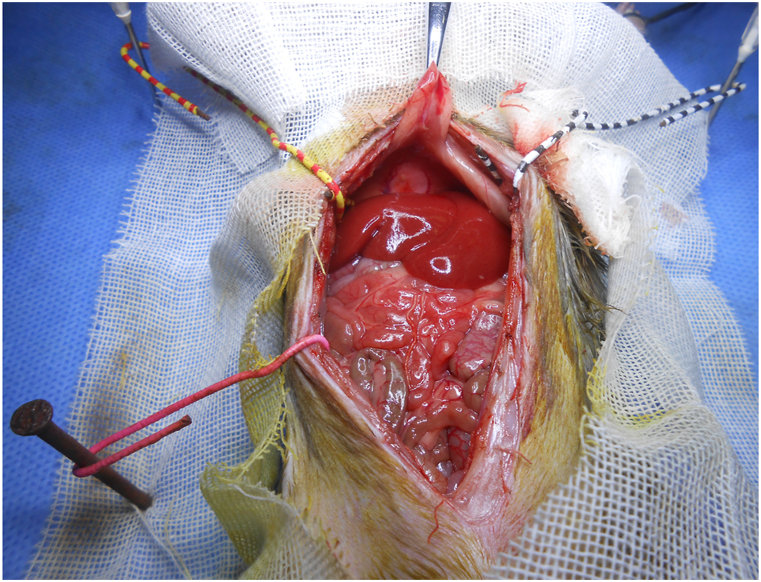
Midline incision for OLT. It was poor for exposure and performance.

**Fig. 3:**
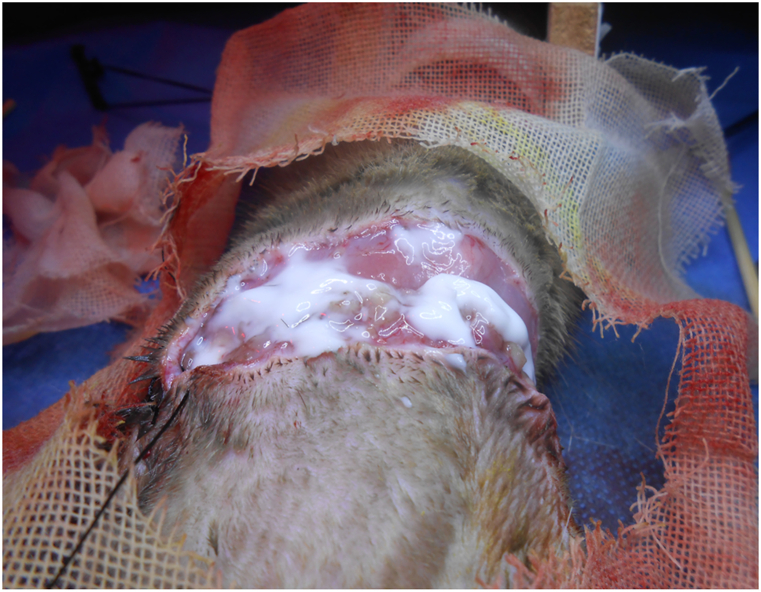
Incision closure.

We made a few modifications to employ inhalation anesthesia of isoflurane, and we made a transverse incision to enter the abdominal cavity for all donor rats. Microsurgeon flushed the donor liver first through the aorta artery with heparinized normal saline (250 u/ml) and re-flushed with 4 °C lactated Ringer's solution through the portal vein (PV), then the donor liver was explanted out and kept in 4 °C lactated Ringer's solution for cuff preparation. Cold ischemia time was about 3 h for all cases. For the recipients of transverse group, we opened the abdominal cavity via the transverse incision and exposed the operation field well with a mosquito to retract the xyphoid cephalad (Fig. 1); the midline incision was made and exposed by retracting the rib arches of two sides for the midline group (Fig. 2). The host native liver was removed and the donor graft was placed orthotopically, we anastomosed the suprahepatic vena cava with 8-0 polypropylene running suture using one-point technique, andin sequence reconnected PV and the infra-hepatic vena cava with the cuff, the anhepatic time was generally in 20 min, and the hepatic artery was reconstructed with a self-made stent [9], the bile duct was reconnected with a stent too. After the muscle layer was closed and coated with lidocaine cream, the skin was closed with a continuous suture (Fig. 3). After cessation of isoflurane inhalation, the recipient rat woke and moved around in a cage under an infrared light for warmth. The regular food and bottle water were available, and 10% glucose solution was additionally supplied for first 4 days, and subcutaneous injection of antibiotic (ceftriaxone) was administered ly once a day, 4 times in total. The recipient stem cells were mobilized with recombinant human granulocyte colony stimulating factor; we subcutaneously injected cyclosporine A at 2 mg/kg once daily for 10 days and ceased afterwards. The detailed protocol of tolerance induction was out of scope here. The severity of self-biting was classified with numerical rating scale and partially modified from the clinical setting: 0 = no evidence of biting, 1 = mild injury (erythema, edema), 2 = moderate injury (minor tissue loss), 3 = severe injury (abdominal organ exposed e, animal requires euthanasia) [10]. The recipient rat alive for more than 3 days was only taken as surgical success and included in this study.

### 1.3 Statistical analysis

The comparisons of incision-related self biting morbidity or mortality between 2 groups were made with the chi-squared test or Fisher exact test; the cumulative survival rates of 2 groups were evaluated with the Kaplan-Meier Curve, the analysis was made with GraphPad Prism 8, and *P* < 0.05 was considered significant statistically.

## 2 Results

**Fig. 4:**
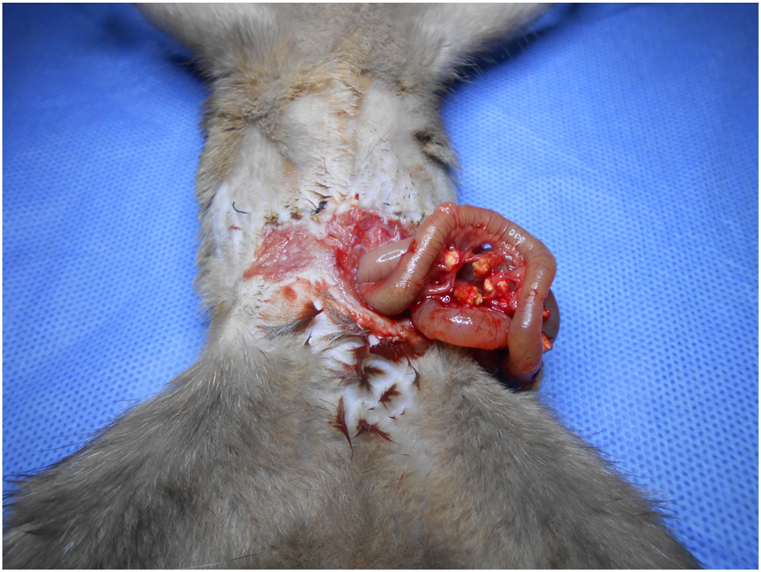
Severe self-biting (degree III) due to transverse incision.

**Fig. 5:**
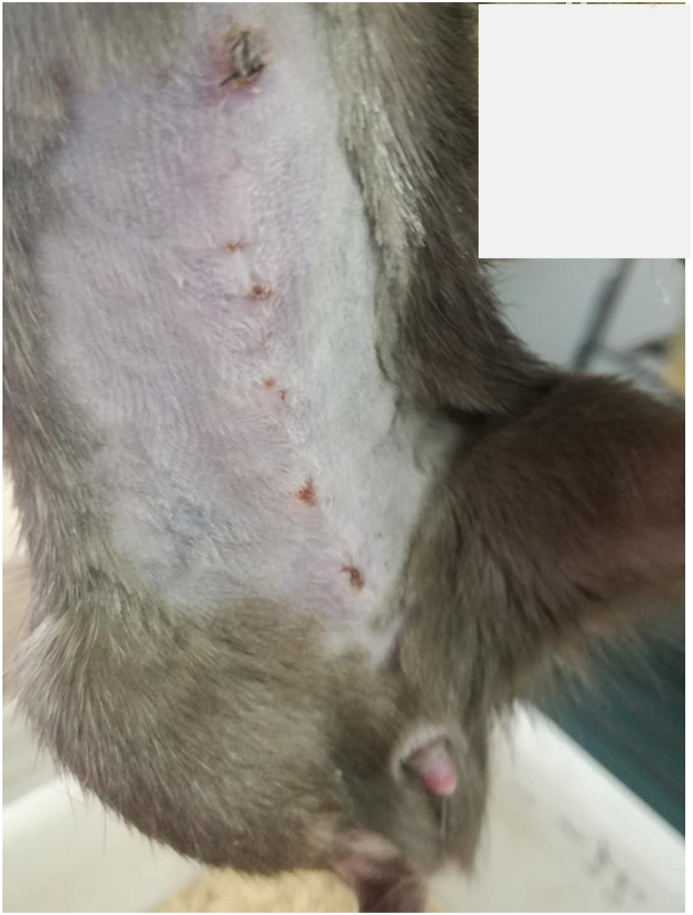
Better healing due to the midline incision for OLT.

**Fig. 6:**
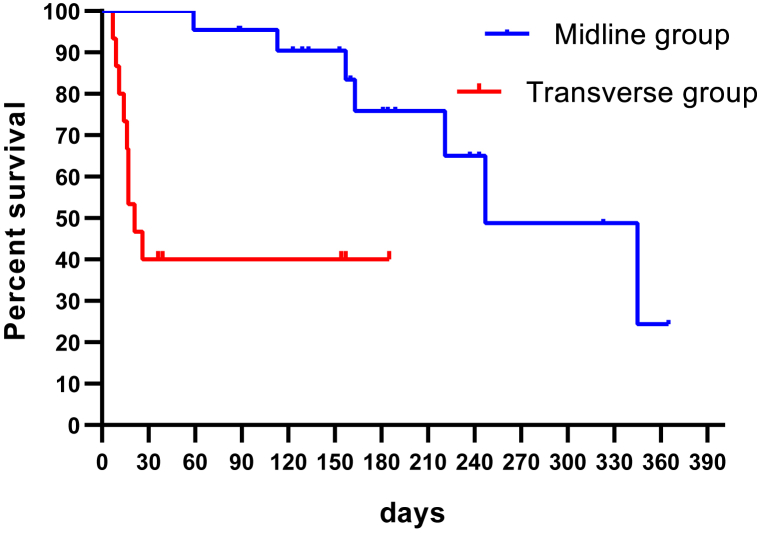
Survivals for rat OLT with the transverse incision or the midline one.

The rat recipient was closely monitored by one microsurgeon. For the transverse incision group, 30-day survival rate was 40% (6/15) (Table 1), self-biting occurred in 13 cases in 7–39 days, the degree 1 of biting occurred in 1 cases, the degree 2 in 2 cases. The degree 3 in 10 cases (Fig. 4), which caused death or euthanasia, the self-biting rate was 86.7% (13/15), For the midline incision group, 30-day survival rate was 100% (22/22), the degree 1 of self-biting occurred in 3 cases, no severe self-biting occurred (Fig. 5). There was significant difference for survival (*p* = 0.0003) and for self-biting rate (*p* < 0.01) between two groups (Fig. 6).

**Table 1:** **Survival time and self-biting occurrences for two groups.** There were significant differences between two groups, ^†^for survival time (*p* = 0.0003), * for self-biting occurrence (*p* < 0.01).

	Transverse incision group (15)	Midline incision group (22)
Self-biting*		
I	1	3
II	2	0
III	10	0
Survival^†^ (days)	21,26,154,9,11,157,7,36,37,39,185,16,17,18,14	153,160,189,243,237,345,365,323,113,153,129,123,163,133,157,89,88,59,184,181,221,247

## 3 Discussion

OLT is a complex surgery including the host liver removal and the donor liver implantation, good exposure is prerequisite. Clinically the subcostal incisional line is below the bilateral anterior rib arches and extended up at a ventral midline in transplant centers which looks like the “Mercedes-Benz logo”, the incision surely transverses the muscle layers and nerves, this contributes to intense postoperative pain and incisional hernia [11,12]. It is equivalent to the transverse incision made for the small animals, at present, the rat, compared to the mouse, is more commonly used for liver transplant tolerance research due to more available combinations of acute or chronic rejection models [[13], [14], [15]], the transverse incision facilitates the surgical procedure to offer better surgical exposure without retraction on both sides of the rib arch, especially when only one surgeon does. In our study, the transverse incision was first and frequently selected because we previouslyperformed in the mouse OLT successfully [9]; different outcomes of OLT for 2 groups were achieved mainly due to incision selections, incision-related injury occurs frequently, especially in BN recipient rats that cannot tolerate the long-term pain and discomfort brought about by greater trauma associated with the transverse incision. The unplanned outcomes drove us to change incision, we selected the midline incision which provided relatively poor exposure, and surgical performances improve with increased experiences. According to duration and distress, OLT is in the one of the most extensive abdominal operation pains. Pain after operation or trauma is unavoidable and its intensity or rating varies depending on many factors including operation performance, overall body conditions, species and age and so on, ongoing pain, discomfort or distress will have negative impact on surgical recovery and life quality. Appropriate pain control can confer on comfort, which, in turn is beneficial to agitation and anxiety reduction, decreasing oxygen consumption and better recovery [16]. For the small animals used, pain management is indispensable and should follow the animal welfare. In fact, diverse animals are susceptible to stress, pain and other discomforts [17]. BN rats should be preferably addressed by microsurgeons to timely pain control, and less injurious incision should preemptively be selected. Clinically, incisional complications are not rare after OLT [18]. Of note, the midline incision has already been applied to perform human LT with larparoscopic assistance in many centers [19].

## 4 Conclusion

Following rat OLT, incision-related self-biting behavior is mainly due to continuous pain derived from the transverse incision selection; the midline incision effective in reducing this self-injury should be preferably selected.

## Author contribution statement

Gaofeng Tang: conceived the study and re-wrote the draft. Huibo Zhao: Analyzed and interpreted the data, revise the manuscript. Guoyong Chen: Contributed reagents, materials, design the study. Shaotang Zhou: performed experiments, wrote the draft.

## Funding

Supported by Hospital Doctor Initiative Fund.

## Availability of data and materials

All data and materials are available on request.

## Declaration of competing interest

The authors declare the following financial interests/personal relationships which may be considered as potential competing interests: Shaotang zhou reports was provided by 10.13039/501100016330Henan Provincial People's Hospital. Shaotang zhou reports a relationship with Henan Provincial People's Hospital that includes: employment and funding grants. Shaotang zhou has patent pending to n/a. none.
